# Architectural Evolution of UAV Tracking Under Efficiency Constraints

**DOI:** 10.3390/s26123668

**Published:** 2026-06-08

**Authors:** Yuxuan Huang, Dongyu Lu, Xinyi Bo, Xiaolan Xie, Shuiwang Li

**Affiliations:** 1 School of Communication and Information Engineering, Shanghai University, Shanghai 200444, China; hyxhuang@yeah.net; 2 College of Computer Science and Engineering, Guilin University of Technology, Guilin 541006, China; 15090250932@163.com (D.L.); bxy51631@gmail.com (X.B.)

**Keywords:** UAV tracking, Transformer, state-space model, Mamba, efficiency, onboard deployment, review

## Abstract

UAV tracking is important for aerial surveillance, inspection, and autonomous perception, yet its progress is constrained by the tension between tracking robustness and limited onboard computation. Compared with existing UAV tracking surveys, this review examines UAV tracking from the perspective of architectural evolution under efficiency constraints, and incorporates Mamba- and SSM-based trackers into the analysis. Specifically, this review discusses UAV tracking as a deployment-constrained problem, analyzes CF, Siamese/CNN, Transformer, and Mamba/SSM trackers from a cross-paradigm perspective, and explains how the literature-reported benchmark results should be interpreted under heterogeneous evaluation settings. We then examine how these architectural paradigms, including recent state-space and Mamba-style models, balance representation ability, interaction strength, temporal modeling, and deployment cost under UAV tracking constraints. Finally, we summarize architecture-level trade-offs and outline open problems in preserving local details during sequence modeling, reproducible efficiency evaluation, hardware-aware design, and multimodal UAV tracking.

## 1. Introduction

UAV tracking is a mission-enabling capability for aerial surveillance, search and rescue, traffic monitoring, infrastructure inspection, and low-altitude autonomous perception. These application scenarios do not impose identical tracking requirements. As summarized in Table 1, search and rescue emphasizes small or partially visible targets and real-time response, traffic monitoring requires stable localization under dense urban clutter, infrastructure inspection stresses precision under weak texture and repeated patterns, and low-altitude autonomous perception requires predictable latency for closed-loop control. Therefore, UAV tracking should be evaluated not only by benchmark performance metrics but also by whether a tracker satisfies the robustness and efficiency requirements of its deployment scenario. In such scenarios, tracking is not an isolated vision module but part of an online and closed-loop perception stack whose outputs directly affect downstream decision-making and mission execution. This system role makes UAV tracking fundamentally different from simply transferring generic short-term tracking methods to UAV platforms. In practice, UAV tracking is jointly constrained by robustness requirements and deployment-side budgets, including stable latency, onboard computation, memory footprint, and power consumption. Therefore, the central concern of this survey is not simply which tracker achieves the highest reported benchmark score; it is what each architectural generation gains—and what it gives up—under resource-constrained aerial deployment.

**Figure 1:**
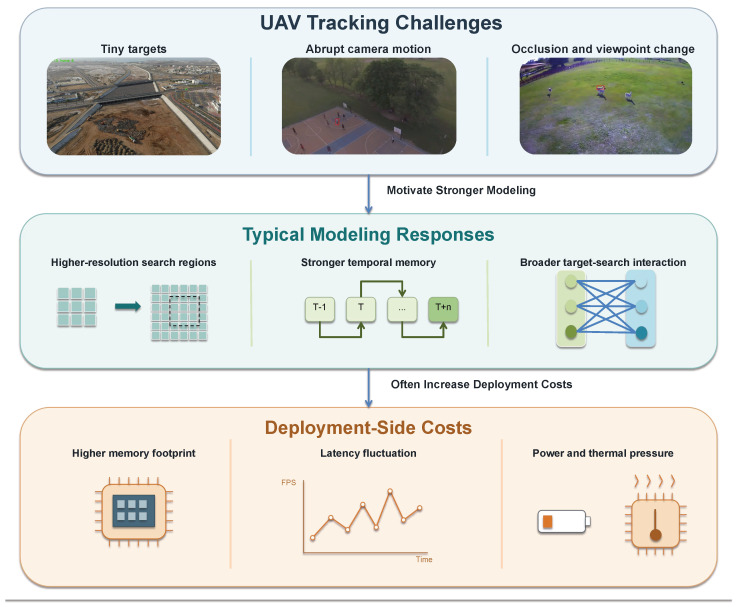
Conceptual illustration of the core trade-off in UAV tracking. Representative UAV challenges, including tiny targets, ego-motion-induced camera movement, occlusion, and viewpoint change, motivate stronger modeling responses such as richer representation, broader context modeling, and temporal memory. These responses may introduce deployment-side costs, including memory demand, latency variability, and power or thermal burden. The arrows indicate typical pressure relationships rather than strict causal correspondences. The red box denotes the target being tracked in the UAV scene.

**Figure 2:**
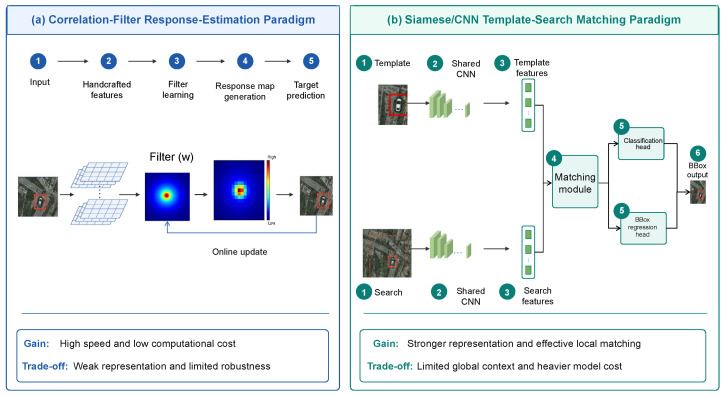
Conceptual summary of the transition from correlation-filter response estimation to CNN/Siamese template–search matching in UAV tracking. The figure abstracts paradigm-level mechanisms rather than specific tracker structures, emphasizing the shift from efficient response estimation to learned representation and matching under efficiency–robustness trade-offs.

**Figure 3:**
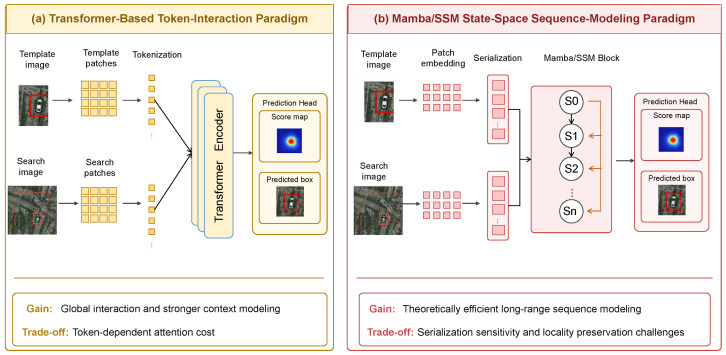
Conceptual summary of the transition from Transformer-based token interaction to emerging Mamba/SSM-based sequence modeling in UAV tracking. The figure abstracts paradigm-level mechanisms and highlights a design-space change rather than a settled replacement relationship between the two paradigms.

The core difficulty of UAV tracking is that its accuracy requirements and deployment constraints are in structural tension. Compared with generic visual tracking, UAV data more frequently involve small targets, weak appearance cues, severe scale variation, motion blur, cluttered backgrounds, intermittent occlusion, and strong camera–target coupled motion. These characteristics naturally push trackers toward higher input resolution, stronger feature extraction, richer template–search interaction, and more persistent temporal reasoning. Yet onboard deployment imposes hard limits on exactly these directions: resolution cannot increase indefinitely, token count cannot grow without cost, and longer memory or stronger interaction often leads to higher latency, larger memory usage, and less stable runtime behavior. As a result, architecture selection in UAV tracking is constrained not only by representational strength, but also by whether the resulting model can run reliably within real-time edge-side budgets. To make this tension explicit, Figure 1 summarizes a common pressure chain in UAV tracking: representative aerial challenges often require stronger modeling responses, and these responses may introduce additional memory, latency, and power burdens during edge deployment. Figure 1, Figure 2 and Figure 3 should therefore be understood as mechanism-level schematic diagrams rather than reproductions of specific tracker architectures. Their purpose is to compare paradigm-level design logic across architectural families, while the model-specific structures and the literature-reported benchmark results are discussed through representative methods in the following sections.

From this viewpoint, the evolution of UAV tracking is interpreted in this review as a sequence of mechanism-level trade-offs rather than as a simple chronology of increasingly powerful models. In the correlation-filter era, methods such as MOSSE [1], KCF [2], SRDCF [3], and ECO [4] emphasized efficient response estimation, high throughput, and low computational overhead, while relying on relatively limited representations. The CNN/Siamese era, represented by trackers such as SiamFC [5], SiamRPN [6], SiamRPN++ [7], ATOM [8], and DiMP [9], shifted the design emphasis toward learned appearance embeddings, template–search matching, and more discriminative prediction modules, but often at the cost of deeper backbones or more complex update strategies. Transformer-based trackers, including TransT [10], STARK [11], OSTrack [12], MixFormer [13], and SwinTrack [14], further shifted the design focus toward explicit template–search interaction and global token communication. These mechanism-level changes are associated with stronger contextual modeling in difficult scenes, but their practical gains and costs remain dependent on dataset, input resolution, implementation, and hardware settings. Even UAV-oriented lightweight variants such as Aba-ViTrack [15], AVTrack [16], TATrack [17], SGLATrack [18], and ORTrack [19] still need to balance tracking robustness with runtime efficiency and deployment feasibility. In this sense, recent UAV tracking research is better understood not as a race to maximize benchmark performance metrics alone, but as an effort to balance tracking robustness, runtime efficiency, and deployment feasibility under practical constraints. Figure 2 and Figure 3 summarize this architectural evolution, illustrating how different paradigms allocate modeling capacity among response estimation, template–search matching, global token interaction, and state-space temporal modeling.

Recent developments in state-space models (SSMs), especially Mamba-style selective SSMs, have attracted attention for efficient long-range modeling in tracking. General visual backbones such as Vision Mamba [20], VMamba [21], and MambaVision [22], together with tracking-oriented studies such as TrackingMamba [23], TemTrack [24], MCITrack [25], MambaVLT [26], and MSTFT [27], suggest that SSM-based modeling provides another way to examine the trade-off between long-range context modeling and efficiency. However, this line of work is still developing, and its practical value for UAV tracking depends on whether the theoretical scaling advantages can translate into practical gains under real implementation constraints while preserving spatial structure and localization fidelity.

This survey focuses on *initialized visual UAV tracking* under efficiency and deployment constraints. Practical UAV vision systems may include detection, recognition, re-detection, and re-identification modules. These modules can provide target initialization, semantic interpretation, recovery support, or identity verification. However, because they follow different input assumptions and evaluation protocols, this review focuses on initialized UAV tracking, where the target is specified at the beginning, and the main analytical issue is how tracking architectures maintain target localization under UAV-specific robustness and deployment constraints. It does not attempt to exhaustively cover UAV multi-object tracking, anti-UAV tracking, swarm pursuit, or non-visual sensing pipelines. These neighboring tasks differ substantially in input structure, system loop, annotation protocol, and evaluation objectives; merging them into a single discussion would blur the main question addressed here, namely how UAV tracking architectures rebalance representation, temporal reasoning, and runtime feasibility under strict edge-side budgets.

Accordingly, this review should be read as an efficiency-oriented and deployment-aware analysis of UAV tracking rather than as a fully exhaustive catalog of all tracking variants. More concretely, it is organized around three guiding questions. First, what UAV-specific challenges does each architectural generation primarily address? Second, what computational, memory, latency, or robustness costs are introduced in return? Third, how should the literature-reported benchmark results and efficiency metrics be interpreted when reported speed, FLOPs, parameter count, and evaluation settings are heterogeneous across the literature?

The remainder of this article follows the same trade-off perspective throughout. It begins by clarifying the UAV tracking problem setting and the UAV-specific phenomena that distinguish it from generic tracking, then revisits the architectural evolution from correlation filters to CNN/Siamese and Transformer-based trackers, emphasizing what each stage improved and what it made more expensive. It next discusses Mamba- and SSM-based developments as recent attempts to balance broader contextual modeling with computational efficiency. Finally, it examines datasets, metrics, benchmark heterogeneity, and open challenges in fair evaluation, edge-aware deployment, and future UAV-oriented tracker design.

### Positioning of This Review and Selection Protocol

This article is a *structured narrative review* rather than a unified experimental re-evaluation or quantitative meta-analysis. Because the summarized results are collected from existing publications, they may differ in dataset usage, metric definition, hardware platform, precision mode, and inference implementation. Therefore, the reported benchmark results are used mainly as supporting material for architecture-level analysis, while direct numerical comparison is emphasized only when evaluation settings are sufficiently aligned.

This review analyzes the architectural evolution of UAV tracking across CF, Siamese/CNN, Transformer, and Mamba/SSM paradigms. Reported benchmark results are used to illustrate architectural trade-offs under heterogeneous evaluation settings, rather than as the basis for a unified cross-paper ranking.

To make this positioning explicit, Table 2 summarizes the focus of this review relative to several common emphases in UAV tracking surveys. The table is intended to clarify the scope and analytical focus of this review.

The review primarily covers papers published from approximately 2015 to early 2026 because this period spans the transition from correlation-filter and Siamese-style trackers to Transformer-based trackers and more recent state-space or Mamba-style models. Earlier general tracking papers are retained when they provide essential methodological background for later UAV tracking developments, while very recent preprints are used selectively to characterize recent architectural tendencies. To avoid overstating early-stage results, preprints are not used as the sole basis for strong conclusions about practical performance or deployment advantages.

The literature search focused on representative UAV tracking studies with commonly used benchmark results, rather than on an exhaustive collection of all related papers. As summarized in Table 3, we focused on papers from approximately 2015 to early 2026 and used Google Scholar as the primary search source, with cross-checking through publisher platforms and digital libraries such as IEEE Xplore, ACM Digital Library, ScienceDirect, SpringerLink, MDPI, and arXiv. Search terms combined UAV tracking-related keywords with architectural and deployment-oriented terms, including “UAV”, “UAV tracking”, “Transformer tracker”, “Mamba tracking”, “state-space model tracking”, and “edge deployment”.

We prioritized studies that were relevant to the architectural evolution of UAV tracking and reported results on widely used UAV tracking benchmarks or useful deployment-related evidence. As summarized in Table 3, after broad preliminary retrieval, duplicate removal, and title–abstract screening, 33 benchmark-relevant tracker studies were selected as candidate studies for detailed full-text assessment. These 33 studies do not represent all papers retrieved during the initial search, but the subset that was sufficiently aligned with initialized UAV tracking and contained benchmark- or deployment-related evidence relevant to the analytical scope of this review. Among them, 20 primary benchmark-relevant studies were retained for the main benchmark-oriented analysis, while 13 were excluded or used only as contextual references after full-text checking because of task mismatch, insufficient comparable results, or validation only on self-collected or special-purpose datasets.

Based on the above positioning and selection protocol, the main contributions of this review are summarized as follows:

- It reframes UAV tracking as a deployment-constrained tracking problem, where accuracy is analyzed together with latency, memory use, runtime stability, and onboard feasibility.
- It provides a cross-paradigm analytical view of CF, Siamese/CNN, Transformer, and Mamba/SSM trackers, focusing on how each architectural family reallocates modeling capacity, interaction strength, temporal reasoning, and computational cost under UAV constraints. In particular, Mamba/SSM-based tracking is reviewed as an emerging design direction rather than a mature replacement for lightweight Transformer designs.
- It clarifies how the literature-reported benchmark results should be interpreted under heterogeneous evaluation settings by using benchmark tables for architecture-oriented interpretation rather than as a strict leaderboard. This helps relate reported accuracy, FPS, FLOPs, and parameter counts to architecture-level design choices without relying on direct cross-paper numerical ranking.

## 2. Problem Setting and UAV Tracking Challenges

### 2.1. Scope and Problem Setting

UAV tracking concerns the online visual localization of a designated target across video frames captured from airborne platforms. In this review, we focus on the initialized setting, where the target is specified in the first frame and then localized in subsequent frames. When benchmarks such as UAV20L are involved, the discussion is extended to long-sequence effects, including accumulated drift, target disappearance, recovery difficulty, and template-update stability. Nevertheless, our focus remains on UAV tracking rather than a comprehensive treatment of explicit re-detection or generic long-term tracking frameworks.

In standard initialized UAV tracking benchmarks, the target is usually specified in the first frame by a bounding box, and the tracker estimates the target location in subsequent frames. In practical UAV perception systems, however, this initial target may be provided by a detector, a recognition module, or human-in-the-loop target selection. Detection localizes candidate objects, recognition or task-specific selection identifies the object of interest, and the resulting bounding box is then used to initialize the tracker. Re-detection and re-identification may further support target recovery after disappearance, occlusion, or identity ambiguity. These modules are therefore important in real UAV perception pipelines, but they follow different input assumptions and evaluation objectives from initialized UAV tracking. For this reason, this review acknowledges their supporting roles while focusing on how tracking architectures maintain the location of a designated target under UAV-specific robustness and deployment constraints.

Within this scope, UAV tracking differs from generic short-term tracking because it is shaped not only by target appearance changes but also by UAV viewpoint variation, platform-induced motion, and the hardware limitations of UAV platforms.

### 2.2. Scene-Level Tracking Challenges and Deployment Constraints in UAV Tracking

This subsection links UAV tracking challenges to later architectural choices. We group the challenges into six dimensions: weak target evidence, geometric instability, temporal discontinuity, occlusion and distractor ambiguity, environmental degradation, and deployment pressure. These dimensions explain why representation quality, template–search interaction, temporal stability, and efficiency-oriented design must be considered together in UAV tracking.

**Tiny targets and weak appearance cues.** One major difference between UAV tracking and generic short-term tracking lies in target scale. UAV platforms frequently observe persons, vehicles, or other objects from elevated viewpoints, causing the target to occupy only a small fraction of the image. This weakens appearance cues, increases vulnerability to background clutter, and makes localization errors more damaging, a difficulty also emphasized by DTB70 [28]. More importantly, tiny targets reduce more than localization precision; they also make the tracker more likely to drift toward visually similar background regions or distractors once weak target evidence is overwhelmed. The problem is further amplified by motion blur, compression artifacts, and illumination variation, and becomes even more severe in nighttime aerial scenarios, as illustrated by UAVDark135 [29]. Therefore, later discussions on lightweight design, token reduction, or feature compression must consider not only whether computation is reduced, but also whether fragile target evidence is unintentionally discarded.

**Scale variation, viewpoint change, and geometric instability.** In addition to being small, UAV targets often undergo rapid scale variation, aspect-ratio change, and viewpoint deformation because both the target and the aerial platform may move simultaneously. A vehicle, pedestrian, or ship may change from a compact blob-like region to an elongated or partially visible region within a short temporal interval. Such geometric instability makes fixed-size search regions, rigid template assumptions, and single-scale feature representations less reliable. It also increases the risk that a tracker will preserve background context while losing the true target boundary. For this reason, UAV tracking requires not only stronger appearance representation, but also scale-sensitive localization, multi-resolution feature preservation, and robust template–search alignment under changing viewpoints.

**Camera–target coupled motion, low frame rate, and large displacement.** A further defining challenge is the strong coupling between platform motion and target motion. In conventional ground-camera tracking, apparent target displacement is often dominated by object motion. In UAV scenarios, however, the ego-motion of the aerial platform itself contributes substantially to apparent displacement, rapid scale variation, perspective deformation, and even partial target disappearance, as documented in UAV123 [30]. As a result, target trajectories often become highly irregular, while viewpoint changes and motion uncertainty are significantly amplified. This problem becomes especially severe under low-frame-rate or large-displacement conditions, where trackers that rely heavily on smooth local search or stable-template assumptions are more likely to fail [31]. This is precisely why benchmarks such as UAV123@10fps and DTB70 are important: they test whether a tracker can tolerate broken temporal continuity rather than merely benefit from it [28,30].

**Occlusion, distractors, out-of-view events, and background clutter.** Another major challenge is the high prevalence of occlusion and background interference, which is also highlighted by DTB70 [28]. UAV videos frequently contain visually similar distractors, repeated man-made structures, vegetation, shadows, roads, rooftops, and low-resolution clutter. Occlusion may arise not only from physical obstacles but also from abrupt camera motion and viewpoint changes that expose confusing background regions. In more difficult cases, the target may temporarily leave the field of view or become nearly indistinguishable from nearby objects. Under such conditions, the key issue is not only whether a tracker remains accurate during easy uninterrupted segments, but whether it can quickly return to the correct target after occlusion, ambiguity, or temporary disappearance. Once an incorrect target region is incorporated into the memory or template state, the resulting error can accumulate into persistent drift. Consequently, UAV tracking evaluation should consider recovery ability, resistance to memory contamination, and re-localization robustness, rather than relying only on small frame-wise precision gains on clean segments.

**Environmental degradation and UAV imaging noise.** UAV tracking is also affected by imaging conditions that are less emphasized in many generic short-term tracking settings. Aerial videos may suffer from illumination variation, low-light or nighttime scenes, haze, shadows, camera vibration, motion blur, compression artifacts, and sensor noise. These degradations weaken target boundaries and further reduce the reliability of local appearance cues. They are especially harmful when combined with tiny targets, because even small amounts of blur or compression may remove discriminative details from the target region. Therefore, robustness in UAV tracking should not be evaluated only through appearance variation in clean daytime scenes, but also through the ability to preserve target evidence under degraded UAV imaging conditions.

**Runtime, memory, and hardware constraints.** Finally, UAV tracking is studied under much stricter deployment constraints than generic tracking, a point already emphasized by ARCF [32]. Although high benchmark accuracy on high-end GPUs is informative, practical UAV deployment requires predictable latency, bounded memory usage, and stable long-duration operation under limited compute and power budgets [31]. In this context, average FPS alone is insufficient: edge-side performance is also shaped by latency fluctuation, operator support, memory footprint, and thermal-induced frequency variation. Accordingly, the most suitable tracker for UAV applications is often not the one with the highest isolated benchmark score, but the one that achieves a reliable balance among accuracy, throughput, resource consumption, and deployment feasibility. This tendency is increasingly reflected in recent UAV-oriented trackers such as TATrack [17], SGLATrack [18], and ORTrack [19].

These challenge dimensions provide the problem basis for the architectural discussion that follows. From correlation filters to Siamese/CNN and Transformer-based trackers, later architectures generally introduce stronger feature representation, richer template–search matching, or broader contextual modeling to handle small targets, occlusion, distractors, and unstable motion in UAV tracking. However, these improvements are often accompanied by higher computation, larger memory use, or less stable latency, especially when high-resolution inputs, dense token interaction, or complex update modules are used. This trade-off also motivates the discussion of Mamba- and SSM-based trackers, which revisit the balance between contextual modeling and deployment-sensitive efficiency.

## 3. Architectural Evolution Under Efficiency Constraints

Architectural progress in UAV tracking should not be read as a simple sequence of replacements. Instead, it reflects a continuing rebalancing of feature representation, template–search interaction, temporal modeling, and deployment feasibility under efficiency constraints.

The architectural categories in this review are defined according to the dominant tracking mechanism rather than the exclusive presence or absence of a specific component. For example, a Transformer-based tracker may still contain convolutional stems, CNN backbones, or convolutional prediction heads, but it is discussed under the Transformer-based paradigm when attention-driven token interaction is the main mechanism for template–search communication. Similarly, methods outside the Siamese/CNN category may also contain convolutional modules; the Siamese/CNN category here refers to methods whose core tracking pipeline is mainly built on learned convolutional representations and template–search matching. Therefore, the following taxonomy should be understood as a mechanism-level abstraction, not as a mutually exclusive component-level classification.

Under this definition, correlation-filter methods are discussed as response-estimation paradigms, Siamese/CNN trackers as template–search matching paradigms, Transformer-based trackers as token-interaction paradigms, and Mamba/SSM-based trackers as state-space sequence-modeling paradigms. In the following subsections, each stage is examined through the same questions: what UAV-specific difficulties it primarily addresses, what modeling ability it strengthens, what computational or deployment cost it introduces, and under what operating conditions it remains attractive.

### 3.1. Correlation-Filter Response-Estimation Paradigm

The significance of the correlation-filter (CF) paradigm lies in the efficiency baseline it established for real-time visual tracking [1]. At its core, the CF family formulated tracking as efficient response estimation, where frequency-domain computation enabled high throughput using relatively simple features [2]. Later developments addressed several structural weaknesses of early CF formulations. Spatial regularization was introduced to reduce boundary effects [3]; background-aware modeling improved resistance to surrounding distractors [33]; and temporal regularization promoted smoother model evolution across frames without fully sacrificing speed [34]. In UAV-oriented extensions, spatio-temporal regularization was further used to suppress abnormal responses caused by abrupt ego-motion, viewpoint variation, or locally unreliable measurements, as reflected in ARCF [32] and AutoTrack [31].

From a UAV perspective, the practical value of CF tracking is clear. These methods are lightweight, easy to implement, and often capable of real-time execution on modest hardware, which makes them attractive when onboard computation is limited or CPU-side deployment is required. Their strengths also match several engineering priorities in UAV tracking, including stable throughput, compact memory use, and a simple update pipeline. However, this efficiency is accompanied by clear limitations. Because most CF variants rely on handcrafted or weakly expressive representations, their robustness decreases under severe appearance change, heavy clutter, similar distractors, strong deformation, or long-term drift. In this sense, the main limitation of the CF paradigm is not speed but its limited ability to meet the growing representation demands of difficult UAV scenes. Even today, CF trackers may remain useful in extremely resource-constrained settings, but their applicability is limited by complex background interference, strong appearance variation, and weak recovery ability after tracking confidence collapses.

### 3.2. Siamese/CNN Template–Search Matching Paradigm

The transition to deep learning shifted the focus of visual tracking from efficient filter optimization to learned representation design. Within this stage, two major lines became especially influential. The first was the *template–search matching* line, represented by SiamFC [5], SiamRPN [6], and SiamRPN++ [7], where tracking was formulated as similarity matching between a target template and a search region. This line was attractive because it preserved a streamlined inference pipeline while improving representation generalization compared with shallow CF methods. The second was the *discriminative estimation* line, represented by ATOM [8], DiMP [9], and PrDiMP [35], which placed greater emphasis on target-state estimation, online adaptation, and discriminative prediction. In mechanism terms, the former emphasized efficient matching, whereas the latter invested more in adaptive prediction and target-state reliability.

For UAV deployment, the template–search line was especially influential because it offered a practical balance between tracking robustness and inference efficiency. For UAV tracking, this paradigm aligned naturally with the initialized tracking setting: a target template is given in the first frame and reused to locate the target in subsequent frames [5]. By reusing the designated target template and avoiding full model updates during inference, Siamese-style tracking often achieved a favorable balance between robustness and runtime [6]. This made it more suitable for deployment than trackers that rely heavily on sensitive online model updates [7]. At the same time, UAV-oriented refinements increasingly focused on preserving this efficiency while coping with small targets, large-scale variation, and edge-side constraints [36]. Feature aggregation strategies improved sensitivity to target scale changes in UAV scenes [37], while compact architectures produced through neural architecture search or lightweight backbone design made mobile deployment more realistic [38]. In practical terms, these design choices can be viewed as UAV-oriented responses to weak target evidence and limited onboard computation, rather than as generic model simplifications [39].

However, the CNN/Siamese paradigm also exposed limitations that were difficult to fully resolve. Template–search communication was often local, implicit, or handled through relatively simple matching operations. As a result, severe occlusion, out-of-view motion, and visually similar distractors could still induce identity drift or make recovery difficult once the target was lost. In many variants, template update and recovery behavior also remained partly heuristic, making long-sequence stability difficult to guarantee. Moreover, as backbones deepened and auxiliary heads became more sophisticated, the speed advantage that made early Siamese tracking attractive for UAV deployment gradually weakened. These limitations motivated later trackers to strengthen cross-region interaction and temporal evidence integration, which became central issues in Transformer-based tracking.

### 3.3. Transformer-Based Token-Interaction Paradigm

The main conceptual shift introduced by Transformers was not simply the use of stronger backbones, but a more explicit formulation of template–search interaction. Compared with Siamese/CNN trackers, which mainly rely on correlation-based matching or local convolutional feature propagation, Transformer-based trackers use attention mechanisms to model interactions between template and search features more explicitly. Early representative models such as TransT [10] showed that template–search fusion could be formulated through attention rather than heuristic matching alone, while later models such as STARK [11], OSTrack [12], and MixFormer [13] moved further toward unified token processing and one-stream interaction. The significance of this transition lies in its simplification of interaction design: instead of manually specifying how template and search features should be fused, the model learns a more direct communication pattern between target cues and search context.

For UAV tracking, this shift was naturally attractive. Broader spatial interaction is useful when the target is small, the background is cluttered, and the relevant evidence is distributed across a wider region than local convolution can reliably integrate. Explicit template–search communication can also improve ambiguity resolution when viewpoint variation and distractors weaken purely local matching. However, these gains come with more serious deployment costs than in earlier architectural stages. The issue is not only the formal quadratic complexity of self-attention, but also the way token growth interacts with UAV tracking requirements. Preserving small-target detail often requires relatively high-resolution search regions, and higher resolution directly increases token count, memory use, latency, and energy demand. As a result, the practical efficiency of Transformer trackers in UAV settings is strongly shaped by input resolution, precision mode, and inference stack, which also helps explain why the literature-reported speed numbers are often difficult to compare directly across papers.

These pressures have pushed recent UAV-oriented Transformer studies toward more concrete efficiency strategies rather than a simple race toward deeper vision Transformers. One line focuses on *structured token or computation reduction*. Aba-ViTrack reduces redundant interaction through background-aware token pruning [15], whereas AVTrack emphasizes adaptive depth and dynamic computation [16]. SGLATrack [18] makes this tendency more explicit by arguing that lightweight ViT trackers still contain substantial layer redundancy; it therefore disables representation-similar layers while retaining a single representative layer, showing that efficient UAV tracking can benefit not only from token sparsification but also from depth-side structural adaptation. LGTrack [40] extends this direction by coupling computation reduction with a robustness-oriented branch: beyond skipping redundant Transformer computation, it introduces Global Grouped Coordinate Attention and occlusion-oriented representation refinement, thereby linking efficiency design more directly to UAV tracking failure cases such as dense clutter and frequent occlusion. Related ideas have also begun to extend beyond standard daytime RGB settings. For example, DARTer [41] combines dynamic feature blending with dynamic ViT activation to reduce redundant computation in low-light UAV tracking, whereas DPTracker [42] strengthens feature encoding through illumination-aware and viewpoint-aware prompt conditioning. Taken together, these studies suggest that the Transformer stage in UAV tracking is no longer simply about increasing token interaction indiscriminately, but about deciding where interaction is necessary, when computation can be reduced, and which UAV-specific challenges—such as occlusion, viewpoint change, and low-visibility degradation—should be explicitly addressed rather than left to generic attention alone.

### 3.4. Cross-Paradigm Perspective

Viewed together, these architectural paradigms can be read as different mechanism-level allocations of representation, interaction, temporal reasoning, and computational budget in UAV tracking. Correlation-filter methods prioritize efficiency and system simplicity, but their limited representation capacity makes them vulnerable in cluttered or strongly deformable UAV scenes. CNN/Siamese trackers introduce learned representations and template–search matching while preserving a practical online pipeline, yet their interaction is often local and their recovery behavior under severe ambiguity remains limited. Transformer-based trackers make template–search interaction more explicit through token communication, but this design also introduces token-dependent computation, memory demand, and implementation-sensitive latency. Seen from this perspective, Mamba and related state-space models are not a disconnected trend, but a continued attempt to revisit long-range interaction and temporal modeling under a different computational mechanism. However, whether this mechanism leads to a practically more efficient UAV tracker remains implementation-dependent rather than guaranteed.

Therefore, the next section does not discuss Mamba merely as a new backbone component or a generic sequence model. Instead, it examines whether state-space modeling can respond to the UAV tracking difficulties that remain insufficiently addressed by previous paradigms, including weak target evidence, broken temporal continuity, occlusion-induced memory contamination, and deployment-sensitive long-range modeling. Table 4 summarizes the cross-paradigm comparison of UAV tracking architectures under efficiency constraints.

## 4. Mamba/SSM-Based Tracking Under UAV Tracking Constraints

### 4.1. Why Mamba Is Attractive but Nontrivial

Because Mamba/SSM-based UAV tracking is still at an early stage, this review treats it as an emerging design direction rather than a mature replacement for Transformer-based tracking. Its practical value still requires more standardized UAV benchmark evaluation and embedded-device validation.

The motivation for introducing Mamba- and SSM-based tracking should be understood from unresolved UAV tracking constraints rather than from sequence modeling alone. Tiny targets require local detail preservation, camera–target coupled motion breaks short-term temporal continuity, and occlusion or distractor interference can contaminate template or memory states. Although Mamba-style state-space modeling is attractive for propagating temporal and contextual information with theoretically lower scaling costs than dense attention, this advantage does not automatically translate into practical UAV tracking efficiency. Compared with lightweight ViT trackers that already reduce attention cost through token pruning, adaptive depth, dynamic computation, and background-aware token selection, Mamba-based UAV trackers may require additional local modeling, feature fusion, template-update control, reliability modules, or greater network depth to preserve local spatial structure and achieve strong visual representation. Thus, Mamba/SSM-based tracking should be viewed as an emerging design direction whose practical value depends on the complete network depth, auxiliary modules, inference kernels, full tracking pipeline, and hardware implementation, rather than as an automatically more efficient substitute for lightweight ViT-based tracking.

### 4.2. From Sequence Modeling to UAV Tracking Requirements

To bridge the gap between 1D sequence modeling and 2D visual data, early efforts focused on developing general visual backbones. Vision Mamba (Vim) [20] showed that bidirectional Mamba blocks could be adapted to visual representation learning. A key turning point came with VMamba [21], which introduced the 2D cross-scan module (CSM) to traverse feature maps spatially. This step is conceptually important because images do not have a natural sequential order in the same way as language. In other words, VMamba did more than add Mamba blocks to vision; it addressed the structural question of how visual features should be scanned and serialized. This issue is highly relevant to UAV tracking, where the scan or serialization strategy can affect the preservation of local target details, especially for tiny targets [29].

Building on these ideas, MambaVision [22] proposed a hybrid Mamba–Transformer architecture, further suggesting that sequence efficiency alone is insufficient for visual tracking unless locality, hierarchy, and interaction quality are also preserved. Inspired by these general visual advances, tracking-specific studies began adapting Mamba to target localization and state estimation. At a high level, these efforts can be read along two main lines. The first line emphasizes *spatial matching and interaction*, where Mamba is used to improve target–search communication under cluttered backgrounds and ambiguous appearance; TrackingMamba [23] is representative of this direction. The second line emphasizes *temporal memory and context propagation*, where Mamba is used to accumulate multi-frame information rather than process each frame pair in isolation; TemTrack [24] and MCITrack [25] illustrate this tendency. For UAV tracking, the former is especially relevant to distractor suppression and ambiguous localization, whereas the latter is especially relevant to weak target evidence, occlusion bridging, and stability across UAV video sequences.

The state-space formulation has also appeared in broader tracking settings such as vision-language or event-based tracking. However, for the purpose of this review, these examples are mainly treated as possible extensions rather than the main focus. The central issue in *visual* UAV tracking remains more specific: how to use state-space modeling to improve target–search interaction and multi-frame stability while keeping the tracker efficient and deployable under UAV constraints.

### 4.3. How Mamba-Based Trackers Adapt to UAV Tracking Challenges

Recent work does not treat Mamba as a drop-in replacement for attention. Instead, recent Mamba-based tracking studies adapt state-space modeling according to specific tracking requirements, including target–search interaction, temporal memory, small-target detail preservation, and deployment cost. From this perspective, the following categories summarize several common adaptation patterns in recent Mamba-based UAV tracking studies.

#### 4.3.1. Combining Mamba with Attention

A prominent UAV-oriented trajectory combines Mamba with attention rather than discarding attention entirely. In functional terms, attention is often used for fine-grained spatial interaction, target localization, and precise template–search alignment, whereas Mamba is often used for lower-cost context propagation and temporal modeling. In UAV tracking, this design is particularly appealing because a purely Mamba-based pipeline may weaken localization precision if local spatial structure is not preserved carefully, while a purely Transformer-based design may remain too expensive under onboard budgets. SAMViTrack illustrates this tendency by combining Mamba and ViT within a search-region adaptive framework for real-time UAV tracking [43]. The main risk is that spatially important information may be over-compressed during sequence propagation, causing tiny-target localization to degrade even when computation is reduced.

#### 4.3.2. Cross-Frame Context and Memory Modeling

Another recurring pattern uses Mamba to strengthen video-level context accumulation and memory propagation. This is attractive in UAV tracking because short-term target cues may be unreliable under motion blur, viewpoint jumps, low resolution, or intermittent target disappearance. MCITrack [25] exemplifies this line by propagating broader contextual information across the sequence instead of treating frame pairs independently, and MambaVLT [26] reflects a related philosophy in multimodal tracking. However, temporal propagation is not automatically beneficial: a mechanism that stabilizes weak target cues can also propagate errors once the tracker has locked onto the wrong target. For this reason, memory enhancement in UAV tracking is most convincing when it is accompanied by confidence-aware, visibility-aware, or reliability-gated update logic rather than unconditional state accumulation.

#### 4.3.3. Preserving Small-Target Details

A third pattern recognizes that efficient sequence modeling alone is insufficient unless small-target details are explicitly preserved. UAV targets often occupy only a tiny fraction of the frame, and their visual details can be severely weakened by aggressive downsampling, coarse tokenization, or indiscriminate pruning. Accordingly, UAV-oriented Mamba adaptation should be understood not only as sequence modeling, but also as a way to preserve weak target details. Useful mechanisms in this category include reducing overly aggressive downsampling, preserving local tokens around candidate target regions, propagating state across multiple feature scales, and strengthening ROI-sensitive fusion before weak target details are submerged by clutter. UAV-oriented trackers such as MSTFT [27] move further toward spatio-temporal fusion that explicitly protects weak target details under UAV tracking uncertainty.

#### 4.3.4. Robustness and Deployment Trade-Offs

A fourth pattern concerns the balance between robustness and practical deployment. In UAV scenarios, occlusion and abrupt motion are especially damaging because ego-motion amplifies apparent instability and can quickly contaminate the tracker state. Methods such as TemTrack [24] strengthen motion robustness through context-aware temporal reinforcement, while Mamba-based spatio-temporal modeling suggests a plausible route to handling difficult UAV frames more gracefully [27]. However, current studies do not support a categorical claim that Mamba-style trackers are more efficient or more deployable than lightweight Transformer trackers. Although Mamba is motivated by linear-complexity sequence modeling, practical UAV trackers must also preserve local spatial details, maintain precise template–search interaction, and control template or memory updates. Once local enhancement, fusion, attention, or reliability-control modules are added, the end-to-end model may become heavier than highly optimized lightweight ViT trackers. Thus, Mamba opens a possible design space for temporal persistence, broader context modeling, and weak target cue accumulation, but its present value lies in expanding the architecture space rather than proving a universal efficiency advantage.

## 5. Datasets, Evaluation Metrics, and Benchmark Analysis

This section shifts from mechanism-level discussion to benchmark-level interpretation. It examines what representative UAV tracking benchmarks actually test and how reported accuracy, speed, and resource costs should be read in relation to deployment-oriented constraints.

### 5.1. Rules for Interpreting the Literature-Reported Benchmark Results

The benchmark tables in this review follow three interpretation rules. First, direct numerical comparison should be treated cautiously because reported results may differ in dataset split, metric definition, input setting, hardware platform, precision mode, and inference implementation. Second, values collected from different publications or later comparative studies are treated as the literature-reported evidence rather than results from a unified re-evaluation. Third, FPS, FLOPs, and parameter count describe different aspects of deployment cost and should not be reduced to a single efficiency score.

Accordingly, the benchmark tables are not used to construct a strict leaderboard or to infer architectural progress from isolated numerical gains. Instead, they are used to support a problem-driven and architecture-oriented interpretation. This review first considers the practical challenges faced by UAV tracking, such as small targets, ego-motion, occlusion, background clutter, and limited onboard computation, and then analyzes how different architectures balance representation quality, robustness, temporal modeling, and deployment cost. The reported benchmark values are then used as descriptive evidence showing how different architectural choices are reflected in robustness, speed, resource cost, and benchmark coverage. Therefore, these tables should be read as evidence for architecture-related reporting patterns and deployment trade-offs, rather than as a basis for strict cross-paper ranking.

### 5.2. Benchmarks and Evaluative Scope: Beyond Simple Accuracy

The UAV tracking domain relies on several core datasets, each engineered to expose different algorithmic weaknesses. Understanding the evaluative intent of a dataset is crucial because the same tracker may behave very differently when the dominant pressure shifts from low-frame-rate displacement to urban clutter, tiny objects, or long-term recovery. A concise summary of representative UAV tracking datasets is provided in Table 5.

**Figure 4:**
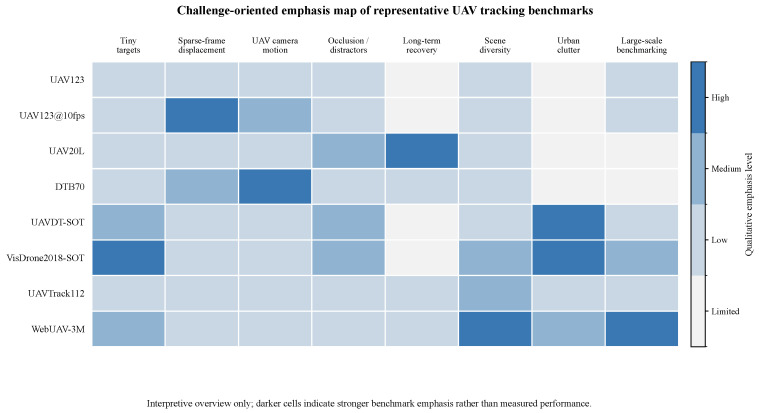
Challenge-oriented emphasis map of representative UAV tracking benchmarks. Rows denote datasets and columns denote challenge dimensions. The emphasis levels are qualitative annotations based on dataset descriptions, annotated attributes, evaluation settings, and common usage in the UAV tracking literature; darker cells indicate stronger relative emphasis. The map supports benchmark interpretation rather than providing a quantitative measurement of dataset difficulty or tracker performance.

Table 5 and Figure 4 shift the dataset discussion from simple listing to challenge-oriented interpretation. Different UAV tracking benchmarks emphasize different failure modes: UAV123 provides a broad general benchmark, UAV123@10fps stresses low-frame-rate displacement, UAV20L highlights long-term drift and recovery, DTB70 focuses on camera-motion instability, UAVDT-SOT and VisDrone2018-SOT emphasize urban clutter and tiny targets, while UAVTrack112 and WebUAV-3M expand scene diversity and scale. The emphasis levels in Figure 4 are qualitative annotations rather than statistically computed difficulty scores; they are assigned according to benchmark descriptions, dataset construction characteristics, dominant evaluation purposes, and challenge attributes emphasized in the original dataset papers. Therefore, benchmark results should be interpreted according to dataset-specific challenge emphasis rather than averaged mechanically across datasets.

### 5.3. Architecture-Oriented Interpretation Across Representative Benchmarks

For readability and cross-era comparability, the panorama tables below do not attempt to include every benchmark listed in Table 5. Instead, they retain benchmarks with comparatively wider reporting coverage across architectural generations, while datasets with more selective or recent usage, such as UAV123@10fps, UAV20L, and UAVTrack112, are discussed separately in the later frontier-oriented tables.

Table 6 and Table 7 aggregate representative methods across major architectural families to organize the types of robustness and efficiency evidence reported in the literature, and they are intended to support architecture-level interpretation rather than strict row-by-row ranking. For these tables, the citation next to each method name denotes the original method paper, while the numerical values are summarized from the unified comparative table reported in SAMViTrack [43], rather than obtained by re-running the trackers in this review. This is particularly important for early CF and CNN/Siamese trackers, because some benchmark scores on later UAV tracking benchmarks, such as WebUAV-3M, were reproduced from later comparative evaluations rather than from the original tracker papers. In Table 7, the asterisk indicates SA-enhanced variants evaluated in SAMViTrack, rather than separate original tracker papers. SAMViTrack reports evaluations on a PC equipped with an Intel i9-10850K CPU (Intel Corporation, Santa Clara, CA, USA), 16 GB RAM, and an NVIDIA TitanX GPU (NVIDIA Corporation, Santa Clara, CA, USA); therefore, the FPS values in these two tables should be interpreted as the literature-reported GPU/CPU speed results under the SAMViTrack evaluation setting, not as directly comparable measurements from a unified re-implementation. Accordingly, the following discussion uses these values to relate architectural design choices to reported robustness and efficiency evidence, rather than to compare small numerical gains or losses among individual trackers. Architecture-level statements in this review are based primarily on mechanism analysis, while the literature-reported benchmark results are used only as supporting evidence; therefore, the cross-paradigm statements in this section should be read as qualitative architecture-level interpretations supported by reported evidence, rather than as statistically tested trend claims under unified experimental conditions.

Taken together, Table 6 and Table 7 summarize how different architectural families are supported by the different types of the literature-reported evidence. For CF trackers, the most informative evidence is not the exact cross-dataset accuracy difference, but their consistently reported CPU-side speed and simple frequency-domain response-estimation mechanism, which explain why they historically served as efficiency-oriented baselines for UAV tracking. For CNN/Siamese trackers, the reported values illustrate the shift from shallow response estimation to learned template–search matching; the key observation is the change in representation and matching mechanism, rather than the precise numerical gap between individual trackers. For ViT-based trackers, the reported FLOPs, parameter counts, and FPS values are mainly useful for understanding why explicit efficiency-control mechanisms, such as token pruning, adaptive depth, lightweight backbones, and dynamic computation, became necessary once global template–search interaction was introduced. Thus, the tables are used to connect architectural mechanisms with the types of robustness and efficiency evidence reported in the literature, not to claim that one tracker is universally superior to another under heterogeneous evaluation settings.

### 5.4. Generic Deep Baselines in UAV Benchmark Evaluation

This subsection complements the preceding architecture-evolution discussion by showing how generic deep trackers have served as baseline references and technical starting points in UAV benchmark evaluation. Many methods evaluated in UAV tracking benchmarks were originally developed for generic visual tracking, but their backbones, interaction patterns, update logic, and deployment simplifications have repeatedly influenced UAV tracking research. Table 8 is therefore included for diagnostic purposes rather than as a separate survey of generic tracking methods. It summarizes representative generic deep trackers frequently used in UAV benchmark evaluation and clarifies the technological starting points from which UAV-specific adaptation has emerged.

In Table 8, the citation next to each tracker name denotes the original tracker paper, while the benchmark and GPU FPS values are reproduced from later UAV tracking comparative studies rather than necessarily from the original tracker papers. Specifically, the DTB70 entries are reproduced from Aba-ViTrack [15], the VisDrone2018 entries from ORTrack [19], and the UAVDT entries from SAMViTrack [43]. These source studies report GPU speed or FPS in their corresponding comparison tables. Aba-ViTrack, ORTrack, and SAMViTrack report evaluations on a PC equipped with an Intel i9-10850K CPU, 16 GB RAM, and an NVIDIA TitanX GPU. Because inference settings and implementation details may still differ across trackers and source studies, these speeds should be interpreted as the literature-reported GPU-speed results rather than directly comparable measurements.

Table 8 helps contextualize the gap between generic visual tracking progress and UAV-specific deployment requirements. Strong generic trackers provide useful representation capacity and interaction mechanisms, but UAV scenarios impose additional constraints, including tiny targets, ego-motion, large inter-frame displacement, and strict runtime budgets. Recent UAV tracking research therefore focuses on selectively adapting generic tracking advances to aerial deployment rather than directly transplanting them.

One adaptation route is *lightweight redesign and pruning*, which attempts to preserve the core interaction pattern of strong generic trackers while reducing overhead. SGLATrack [18] is representative here because it explicitly attributes part of UAV tracking inefficiency to redundant Transformer depth rather than to attention alone. A second route is *dynamic computation*, in which model capacity is allocated selectively rather than uniformly across frames or layers. AVTrack is representative of this logic in the main RGB UAV tracking setting, while DARTer [41] extends a related efficiency idea to a separate nighttime UAV tracking branch, where redundant activation becomes even more costly under degraded imagery. A third route is *motion- and occlusion-aware robustness enhancement*, where the architecture is reshaped to better tolerate aerial instability, target disappearance, or distractor interference. LGTrack [40] fits this pattern by combining efficient layer adaptation with dedicated feature enhancement for occlusion-heavy scenes.

A fourth route is *task-aware hybridization*, where stronger global modeling is retained only where it is most useful. SAMViTrack [43] illustrates this tendency by combining Mamba and ViT while using an adaptive search-region mechanism to reduce unnecessary overhead. Read in this way, Table 8 is more than a baseline list; it shows how UAV tracking selectively absorbs generic progress while reorganizing it around redundancy reduction, adaptive computation, robustness enhancement, and deployment-aware hybridization.

### 5.5. The Mamba Frontier: Broadening the Design Space

Table 9 and Table 10 summarize early Mamba/SSM-oriented evidence in UAV tracking. Because this branch is still young, the most informative signals are not isolated best scores, but recurring design patterns and reporting gaps, including spatial–temporal serialization, hybrid Mamba–Transformer interaction, adaptive search regions, and incomplete reporting of hardware, precision, and deployment settings. For these two tables, benchmark values, efficiency indicators, and hardware settings follow the corresponding source papers, and the citation next to each method name denotes the original method paper. The reported values should therefore be interpreted as the literature-reported evidence rather than results obtained by re-running the trackers in this review. TrackingMamba reports training on a single NVIDIA 4090 GPU and testing on a single NVIDIA 4060Ti GPU; SAMViTrack reports evaluation on a PC equipped with an Intel i9-10850K CPU, 16 GB RAM, and an NVIDIA TitanX GPU; MambaNUT reports evaluation on a PC equipped with an Intel i9-10850K CPU, 16 GB RAM, and an NVIDIA TitanX GPU; MSTFT reports experiments on a server with 4 NVIDIA Tesla A100 GPUs and reports a tracking speed of 45 FPS in its source paper; SPM-Track reports speed evaluation on an NVIDIA RTX 4090 GPU and an NVIDIA Jetson AGX Orin platform; and LF-SSM reports evaluation on an Intel i9-13900K CPU, an NVIDIA RTX 4090 GPU, and 64 GB memory, with edge-platform deployment further evaluated on an NVIDIA Jetson Orin Nano using TensorRT FP16 optimization. In Table 10, platform or inference-setting information is indicated in parentheses when reported by the source papers. CPU-only speeds are marked as “CPU”, while Jetson-class values are marked by the corresponding embedded platform and should not be interpreted as CPU-only inference speeds. Because these early Mamba/SSM-oriented studies differ in benchmark coverage, implementation details, hardware platform, precision mode, and reporting completeness, the efficiency values in Table 10 should be used as reference information only and should not be used to rank Mamba-family trackers directly.

Table 10 is not intended to rank Mamba-family trackers by efficiency. Instead, it exposes how uneven current deployment reporting remains: some studies report only speed, some report parameters and FLOPs, and only a few include embedded-device results. Methods without clearly comparable reported efficiency values are omitted from this efficiency table rather than treated as zero-information entries. This incompleteness is itself important evidence for the need to standardize efficiency evaluation in future UAV tracking work.

More importantly, the reported efficiency evidence does not show a uniform practical advantage of Mamba-style UAV trackers over recent lightweight ViT trackers. Some Mamba or Mamba-hybrid trackers report compact complexity and high speed, whereas others introduce substantially larger parameter counts or FLOPs after adding spatio-temporal fusion, local-detail preservation, template-update verification, or context-prediction modules. This suggests that the theoretical linear-complexity property of state-space modeling is only one component of the final efficiency profile. In UAV tracking, end-to-end cost is also shaped by feature extraction, two-dimensional locality preservation, template–search fusion, update reliability, memory access patterns, and hardware kernel support. Therefore, the current Mamba evidence should be read as heterogeneous and exploratory: it indicates possible routes for long-range and temporal modeling, but it does not yet establish Mamba as a consistently lighter or more accurate replacement for the latest lightweight ViT-based trackers.

A related but analytically separate branch is nighttime UAV tracking. Relative to the RGB benchmark axis emphasized in Table 6, Table 7, and Figure 4, dedicated low-light UAV benchmarks probe a different operating regime and should therefore not be merged directly into the main benchmark panorama. Methods such as MambaNUT [69], DARTer [41], and DPTracker [42] are valuable in this survey because they show how efficiency-oriented tracking design extends into low-illumination aerial scenarios. Their role here, however, is supplementary rather than directly comparative: they indicate methodological extensibility, but they do not yet provide benchmark results that are directly commensurate with the main RGB-oriented evaluation track discussed above.

Within the main Mamba-related UAV tracking literature, the currently available evidence points to several emerging routes rather than a single converged recipe. These routes are best interpreted as benchmark-visible design tendencies rather than isolated mechanisms, because they reflect different attempts to balance spatial detail, temporal persistence, and deployment cost. One route is *high-throughput serialization*, where the main objective is to exploit efficient sequence scanning while preserving sufficient spatial detail for tracking. TrackingMiM is representative in this sense, since it combines nested spatial–temporal Mamba scanning with retrieval-augmented tracking attention, thereby making serialization more explicitly target-aware. A second route is *lightweight hybridization*, in which Mamba is combined with Transformer-style visual modeling to balance interaction quality against computational cost. SAMViTrack belongs to this route, suggesting that hybrid backbones can remain competitive when paired with adaptive search-region control. A third route is *reformulated state evolution*, where the contribution is no longer limited to reusing a standard selective scan, but extends to redesigning the state update mechanism itself for deployment-oriented tracking. LF-SSM is notable in this respect because it reports a reformulated state-evolution strategy and additionally includes embedded-device efficiency on a Jetson-series platform.

In this sense, the current Mamba frontier should not be defined only by the slogan of linear complexity. It is increasingly defined by how sequence modeling is serialized, whether hybridization is preferable to full replacement, whether state evolution itself should be reformulated, and under which deployment settings these design choices actually matter.

Overall, the currently available evidence does not support framing Mamba as a universal replacement for Transformers in UAV tracking. A more defensible interpretation is that it expands the present trade-off space by offering new ways to balance sequence length, spatial detail, temporal persistence, and deployment cost. Future work in this line would be substantially easier to interpret if it reported results under closer deployment alignment—ideally with matched hardware, matched resolution, matched precision mode, and matched inference stack—and, when possible, with reproducible accuracy–efficiency curves or a small set of clearly defined operating points.

### 5.6. Insights from the Literature-Reported Benchmark Results

The benchmark results discussed above reveal several unresolved issues. First, efficiency reporting remains difficult to compare fairly across papers because input resolution, hardware platform, precision mode, and deployment toolchain are often inconsistent. Second, aggregate benchmark scores can mask aerial-specific failure modes such as tiny-object collapse, abrupt viewpoint change, and strong ego-motion drift. Third, the field still lacks sufficiently standardized stratified reporting for the scenario slices that matter most in UAV deployment.

These observations imply that the central difficulty in UAV tracking is no longer simply whether stronger global modeling helps in principle. The more practical question is under what efficiency regime such modeling remains worthwhile. Put differently, the field should not expect a single global leaderboard to resolve all design choices. A more realistic objective is to identify task-aware operating points: how much computation, latency, memory, and power are available, and what level of robustness is required against tiny targets, motion stress, and recovery-critical failures.

For this reason, benchmark interpretation in UAV tracking should increasingly move beyond universal ranking. What matters in practice is not only whether a tracker improves aggregate scores, but whether it delivers the right balance of computation, latency, memory, power, and robustness for the deployment scenario at hand. The next section revisits this issue from the perspective of open challenges and future research directions.

## 6. Open Challenges and Future Directions

Although UAV tracking has progressed from correlation filters to CNN/Siamese trackers, Vision Transformers, and more recently Mamba-based state-space models, several fundamental challenges remain unresolved. These challenges are not only inherited from generic visual tracking; they are amplified by the aerial setting, where targets are often small, motion is strongly coupled with camera ego-motion, and deployment is constrained by onboard computation and power budgets. Future research should therefore focus less on directly importing generic backbone advances into UAV tracking and more on *UAV-oriented co-design* across representation, temporal modeling, evaluation, and deployment. Among the many open directions, two priorities appear especially urgent for the next stage of the field: *small-target evidence preservation in sequence modeling* and *reproducible standardization of efficiency evaluation*. Without the former, stronger global modeling may still fail on the most UAV-critical visual cases; without the latter, claimed deployment gains remain difficult to interpret or reproduce.

A first open challenge is *locality-aware sequence modeling for small-target UAV tracking*. In UAV videos, the target often occupies only a tiny fraction of the frame, and its discriminative cues can be overwhelmed by background clutter, compression artifacts, and abrupt viewpoint variation. This means that long-range context alone is insufficient: a UAV tracker must also preserve fine-grained local evidence throughout the feature extraction and temporal propagation process. For Mamba-based designs in particular, this raises a nontrivial question. While state-space models are attractive for efficient long-range dependency modeling, their effectiveness in UAV tracking depends on whether the serialization and state update process can retain small-target details rather than wash them out. Future work should therefore explore locality-aware scan strategies, scale-sensitive token routing, multi-resolution state propagation, and update mechanisms that explicitly protect weak target evidence under large background dominance. In other words, the key issue is not simply whether Mamba can replace attention, but whether Mamba can be redesigned to fit the spatial statistics of UAV tracking. A useful way to verify progress in this direction is to report stratified results by target-size bucket—for example, AUC and Precision on small-target subsets defined by bounding-box pixel area or area ratio—and to test whether different scan or routing strategies measurably reduce small-target loss or distractor takeover.

A second challenge is *robust temporal memory under ego-motion and target ambiguity*. In UAV tracking, temporal instability is not caused only by target motion. Rapid drone maneuvers, changing altitude, camera shake, and viewpoint switching all distort apparent motion patterns. As a result, naive temporal accumulation can easily propagate errors rather than improve robustness. This is particularly relevant to both Transformer and Mamba trackers: stronger temporal modeling is beneficial only when the memory mechanism can distinguish reliable evidence from transient noise. Future research should therefore move toward confidence-aware temporal persistence, where state updates are conditioned on motion stability, target visibility, and background interference. This includes adaptive template refresh, memory gating under abrupt viewpoint change, and recovery-oriented mechanisms for long-term occlusion or target disappearance. For UAV tracking, the goal is not simply to remember more history, but to remember the *right* history. To support this claim empirically, future studies should report metrics that more directly reflect memory reliability, such as recovery time after occlusion, drift rate after ambiguity, and re-localization success under target disappearance.

A third direction is *small-target-centered benchmark design and evaluation*. A persistent limitation in current UAV tracking research is that many trackers are still developed primarily on generic tracking datasets and only later transferred to UAV benchmarks. This often obscures aerial-specific failure modes, including severe scale collapse, low-resolution target appearance, cluttered backgrounds, motion blur, and ego-motion-driven trajectory irregularity. As a result, a tracker may appear competitive in aggregate metrics while still being fragile in the most safety-critical UAV scenarios. Future benchmark construction should therefore place greater emphasis on small-target subsets, motion-intensity stratification, visibility-aware evaluation, and category-specific aerial difficulty analysis. More fine-grained reporting would help distinguish whether a method truly improves UAV tracking robustness or merely inherits generic tracking competence from large-scale pretraining. To make this recommendation more actionable, future work should explicitly define size buckets by pixel area or target-to-frame area ratio, motion bins by inter-frame displacement or motion-energy statistics, and visibility labels by occlusion/out-of-view severity rather than relying only on undifferentiated sequence-level averages.

A fourth open problem is *benchmark standardization for efficiency and reproducible deployment*. The UAV tracking literature increasingly reports FPS, FLOPs, and parameter counts, yet these quantities are still defined under inconsistent settings. Different papers use different input resolutions, hardware platforms, precision modes, software stacks, and acceleration frameworks, making efficiency claims difficult to compare fairly. This problem is especially important in UAV tracking, where deployment feasibility is not a peripheral issue but part of the core research objective. A mature evaluation protocol should therefore specify hardware assumptions, inference precision, resolution settings, and whether optimizations such as TensorRT or platform-specific kernels are used. Beyond reporting a single best number, future work should provide reproducible operating points and matched Pareto curves under standardized hardware settings. For UAV tracking, a method is only fully convincing when its claimed accuracy-efficiency trade-off can be independently reproduced.

Table 11 summarizes a minimum reporting checklist that would make future UAV tracking efficiency claims easier to reproduce and compare. The checklist is deliberately practical: it focuses on information that directly affects whether a tracker can run reliably on edge UAV hardware, rather than on adding decorative statistics.

A fifth direction is *hardware-aware model design for edge UAV platforms*. The historical development of UAV tracking shows that architectural success in this field is tightly coupled with deployability: CF trackers remained influential because they were easy to run onboard, while later lightweight CNN, ViT, and hybrid models succeeded when they narrowed the accuracy–efficiency gap. Future UAV trackers should therefore be designed with the hardware target in mind from the outset rather than optimized for accuracy first and compressed afterward. For Mamba-based UAV tracking, future work should verify whether lightweight state design, selective token/state updates, and operator-level optimization can translate theoretical efficiency into real latency and power advantages on embedded UAV hardware.

A sixth emerging direction is *multimodal and persistent UAV tracking*. As UAV sensing systems become more diverse, future tracking scenarios will increasingly involve multispectral, infrared-visible, event-based, or even language-guided inputs. Recent developments already suggest that multimodal UAV tracking is becoming a realistic research direction rather than a speculative extension. For example, MUST points to the growing relevance of multispectral UAV tracking [72], MambaEvt indicates the potential of event-based sequence modeling [73], and MambaVLT further suggests that state-space modeling can be extended to vision-language tracking settings [26]. In this context, Mamba is particularly interesting because its state-space formulation naturally supports sequential evidence accumulation and may offer a unified mechanism for cross-modal temporal fusion. However, this promise will only be realized if future work addresses modality imbalance, asynchronous sensing, and long-horizon consistency rather than simply concatenating heterogeneous features. For UAV tracking, multimodal tracking should be understood primarily as a way to improve robustness in the scenarios where RGB tracking is weakest—for example, low visibility, night-time observation, severe illumination change, or weak-texture small targets—rather than as a purely task-expansion exercise. At the same time, multimodal gains must still be evaluated under the same deployment budget logic as RGB tracking, because additional sensing and fusion do not remove the need to account for runtime, memory, and power cost.

Finally, the field would benefit from a stronger emphasis on *task-aware operating regimes* rather than universal leaderboard thinking. UAV tracking spans multiple application scenarios, including surveillance, search and rescue, traffic monitoring, and long-range observation, each of which imposes a different balance between precision, robustness, latency, and power consumption. Future studies should therefore clarify which operating regime a tracker is intended for and evaluate it accordingly. For example, search-and-rescue scenarios may value recall, long-horizon stability, and recovery after disappearance more than peak throughput; infrastructure inspection may prioritize predictable latency and sustained low-power operation over maximal benchmark accuracy; and traffic or urban monitoring may require stronger robustness to clutter and distractors under moderate but stable compute budgets. Framing future progress in terms of scenario-specific operating points would make UAV tracking research more practically meaningful and methodologically fair.

## 7. Conclusions

This review examined UAV tracking from an efficiency-constrained perspective and showed that the transition from correlation filters to CNN/Siamese models, Transformers, and more recently, Mamba-based state-space models is better understood as a sequence of architectural trade-offs than as a simple replacement chain. In UAV tracking, accuracy alone is insufficient for judging practical value because latency, resource cost, and deployment conditions jointly determine whether a tracker is truly usable. Current evidence suggests that Mamba should not yet be viewed as a universal replacement for lightweight Transformers; rather, its main significance lies in expanding the design space for balancing temporal modeling, spatial detail, and edge-side efficiency. Importantly, the reviewed efficiency evidence does not show that current UAV-oriented Mamba/SSM trackers are consistently lighter or more deployable than optimized lightweight ViT trackers. In several cases, the auxiliary modules required for locality preservation, cross-frame feature fusion, target-detail protection, or reliable memory control may offset the theoretical sequence-scaling advantage. Future progress in UAV tracking will therefore depend not only on new architectures, but also on more reproducible benchmark interpretation, standardized efficiency reporting, and closer alignment between model design and deployment conditions.

## Figures and Tables

**Table 1:** Representative UAV tracking application scenarios and their differentiated tracking requirements.

Scenario	Dominant Challenges	Robustness Requirements	Efficiency Requirements
Search and rescue	Tiny or partially visible targets, abrupt viewpoint change, occlusion, low-light scenes, and uncertain motion.	Strong target persistence, recovery after temporary disappearance, and resistance to weak appearance cues.	Real-time inference with stable latency to support timely decision-making.
Traffic monitoring	Dense urban clutter, similar vehicles, scale variation, and frequent background interference.	Stable localization and distractor suppression under crowded scenes.	Moderate to high throughput, depending on UAV hardware and monitoring duration.
Infrastructure inspection	Weak texture, repeated structural patterns, viewpoint change, and local deformation.	High localization precision and resistance to repeated-pattern distractors.	Moderate latency may be acceptable, but memory and power consumption should remain bounded for long missions.
Low-altitude autonomous perception	Strong ego-motion, large inter-frame displacement, motion blur, and closed-loop control requirements.	Stable short-term tracking and reliable response under sudden motion changes.	Predictable latency, compact memory use, and low power consumption are critical.

**Table 2:** Positioning of this review relative to common emphases in UAV tracking surveys.

Common Review Emphasis	Typical Focus	Focus of This Review
Tracker-family taxonomy	Organizes UAV tracking methods according to algorithm families, representative trackers, and technical categories.	Examines how different architectural families balance representation ability, robustness, runtime efficiency, and deployment feasibility.
Benchmark results	Summarizes and analyzes the results reported by representative UAV tracking studies.	Uses reported benchmark results as supporting material for architecture-level analysis, while noting differences in datasets, protocols, hardware platforms, input settings, and inference implementations.
General tracking or Transformer-era review	Provides broader background on visual tracking or Transformer-based tracking methods.	Focuses on UAV-specific constraints, including tiny targets, UAV ego-motion, occlusion, cluttered backgrounds, latency, memory use, and onboard computation.
Mamba/SSM-related discussion	Introduces state-space modeling and Mamba-style architectures as recent sequence-modeling techniques.	Discusses Mamba/SSM-based trackers in relation to UAV tracking constraints, including local detail preservation, temporal modeling, implementation cost, and deployment uncertainty.

*Note:* The table clarifies the scope of this review and is not intended to rank or evaluate previous surveys.

**Table 3:** The literature search and screening protocol used in this review.

Item	Description
Review type	Structured narrative review focusing on representative UAV tracking studies with commonly reported benchmark results.
Search period	Approximately 2015 to early 2026, covering the main evolution from CF and Siamese/CNN trackers to Transformer and Mamba/SSM trackers.
Search update	The literature search was last updated in May 2026.
Language range	English-language papers were considered.
Main search source	Google Scholar.
Additional sources	Publisher platforms and digital libraries, including IEEE Xplore, ACM Digital Library, ScienceDirect, SpringerLink, MDPI, and arXiv.
Publication status	Peer-reviewed journal and conference papers were prioritized. Recent arXiv preprints were used selectively as contextual references but were not used as the sole basis for strong conclusions about practical performance or deployment advantages.
Search terms	Combinations of “UAV tracking”, “efficient tracking”, “Transformer tracker”, “Mamba tracking”, “state-space model tracking”, and “edge deployment”.
Candidate studies for full-text assessment	After broad preliminary retrieval, duplicate removal, and title–abstract screening, 33 benchmark-relevant tracker studies were selected for detailed full-text assessment. These studies were the subset most closely aligned with initialized UAV tracking and benchmark- or deployment-related analysis.
Main retained benchmark entries	20 primary benchmark-relevant studies were retained for the main benchmark-oriented analysis covering CF, Siamese/CNN, Transformer, and Mamba/SSM trackers. Additional generic trackers, dataset papers, and recently reported baselines were used as contextual references when needed. For early trackers whose original papers predate later UAV tracking benchmarks, benchmark values were included only when reproduced or re-evaluated in later comparative studies under an initialized tracking protocol, with result sources indicated in the corresponding explanatory text and, where necessary, in table captions.
Inclusion criteria	Papers were included when they reported results on representative UAV tracking benchmarks, introduced influential tracking architectures, provided deployment-related information such as FPS, FLOPs, parameters, or hardware setting, or helped explain UAV-specific benchmark protocols and failure factors.
Exclusion or de-emphasis criteria	Of the 33 candidate tracker studies selected for full-text assessment, 20 were retained for the main benchmark-oriented analysis, and 13 were excluded or used only as contextual references. Papers were excluded or de-emphasized when they were not aligned with the initialized UAV tracking setting, lacked sufficient benchmark or efficiency information, or were validated only on self-collected or special-purpose datasets.
Use of non-comparable studies	Generic tracking baselines, dataset papers, recent preprints, and low-light or multimodal studies were used as contextual references when relevant, but were not included as primary entries in the main benchmark tables.

**Table 4:** Cross-paradigm comparison of UAV tracking architectures under efficiency constraints.

Paradigm	Key Strengths	Key Weaknesses	Computational Requirements	Deployment Risks	Suitability for UAV Edge Devices
Correlation filter	Very fast response estimation, low memory cost, simple optimization, and strong CPU-side practicality.	Limited representation capacity; weak robustness under clutter, deformation, occlusion, and similar distractors.	Low. Many methods can run efficiently on CPUs or modest hardware.	Model drift under complex backgrounds; limited recovery once the response map becomes unreliable.	High for extremely constrained devices, but the accuracy ceiling is limited in difficult UAV scenes.
Siamese/CNN	Good speed–accuracy balance; efficient template–search matching; stronger learned representation than CF trackers.	Mostly local interaction; limited long-term reasoning; template mismatch and heuristic update may cause drift.	Moderate. Cost depends strongly on backbone depth, feature resolution, and prediction head design.	Small targets may be weakened by downsampling; recovery is difficult after occlusion or out-of-view events.	Generally suitable for real-time UAV tracking when lightweight backbones and careful update strategies are used.
Transformer	Explicit template–search interaction; stronger global relation modeling; better ambiguity handling in cluttered scenes.	Token-dependent cost; higher memory demand; latency is sensitive to input resolution and implementation details.	Medium to high. Computation and memory grow with token number, resolution, and attention design.	Unstable latency, high memory use, and hardware-dependent acceleration; aggressive token reduction may remove weak target evidence.	Suitable when lightweight design, pruning, adaptive depth, or dynamic computation is used, but standard Transformer designs may be too costly for small UAV platforms.
Mamba/SSM	Potentially efficient for long-range contextual or temporal modeling; useful for temporal persistence and weak-evidence accumulation, but end-to-end cost is implementation-dependent.	Sequence scanning may weaken local spatial structure; achieving strong performance may require deeper Mamba stacking or additional local modeling modules.	Potentially moderate or low, but actual speed depends on kernels, memory access, precision mode, and hardware support.	Loss of fine target details, state contamination after distractor takeover, and uncertain edge-device acceleration.	Useful for exploring new tracking architectures, but not yet a settled replacement for lightweight Transformer trackers.

**Table 5:** Representative UAV tracking benchmark datasets and their challenge-oriented evaluation focus.

Dataset	Year	Modality/Task	Scale	Main Challenge Emphasis	Suitable Evaluation Focus
UAV123 [30]	2016	RGB, tracking	123 seq., >110 K frames	General UAV tracking with scale variation, viewpoint change, fast motion, and background clutter.	Overall robustness under common UAV tracking conditions.
UAV123@10fps [30]	2016	RGB, tracking	123 seq.	Sparse temporal sampling and enlarged inter-frame displacement.	Robustness to low frame rate, abrupt motion, and broken short-term continuity.
UAV20L [30]	2016	RGB, long-term tracking	20 long seq.	Long sequences with accumulated drift, target disappearance, and recovery difficulty.	Long-term stability, update reliability, and resistance to error accumulation.
DTB70 [28]	2017	RGB, tracking	70 seq., ∼41 K frames	Strong UAV camera motion, motion blur, viewpoint change, and dynamic background.	Motion robustness under platform-induced instability.
UAVDT-SOT [44]	2018	RGB, tracking	50 test seq.	Urban scenes with small objects, similar distractors, and cluttered traffic backgrounds.	Distractor suppression, small-object localization, and urban UAV tracking robustness.
VisDrone2018-SOT [45]	2018	RGB, tracking	132 seq., 106,354 frames	Dense urban clutter, tiny targets, occlusion, and frequent background interference.	Small-target tracking and ambiguity resolution in crowded scenes.
UAVTrack112 [46]	2021	RGB, tracking	112 seq., >100 K frames	Diverse real-world scenes with multiple UAV-specific attributes.	Generalization across broader aerial scenarios.
WebUAV-3M [47]	2023	RGB, tracking	4500 videos, >3.3 M frames	Large-scale UAV tracking with diverse scenes and many videos.	Large-scale training/evaluation, deep tracker generalization, and data-driven robustness.

**Table 6:** The literature-reported benchmark overview of representative CF/CNN trackers evaluated on UAV tracking benchmarks. “P/S” denotes Precision/Success, and “G/C” denotes GPU/CPU FPS; missing entries are marked by “-”. Values should be interpreted according to the rules in Section 5.

Method	Year	Arch	DTB70	UAVDT	VisDrone	UAV123	WebUAV-3M	FPS	FLOPs	Params	Efficiency Strategy
			(P/S)	(P/S)	(P/S)	(P/S)	(P/S)	(G/C)	(G)	(M)	
KCF [2]	2015	CF	46.8/28.0	57.1/29.0	68.5/41.3	52.3/33.1	39.8/21.6	-/468.5	-	-	Frequency-domain CF
fDSST [48]	2017	CF	53.4/35.7	66.6/38.3	69.8/51.0	58.3/40.5	43.5/28.5	-/132.4	-	-	Scale-adaptive DCF
ECO-HC [4]	2017	CF	63.5/44.8	69.4/41.6	80.8/58.1	71.0/49.6	61.3/40.2	-/66.9	-	-	Efficient convolution operators
MCCT-H [49]	2018	CF	60.4/40.5	66.8/40.2	80.3/56.7	65.9/45.7	52.1/34.3	-/55.3	-	-	Multi-cue CF ensemble
AutoTrack [31]	2020	CF	71.6/47.8	71.8/45.0	78.8/57.3	68.9/47.2	56.5/35.6	-/50.7	-	-	Spatio-temporal regularization
RACF [50]	2020	CF	72.6/50.5	77.3/49.4	83.4/60.0	70.2/47.7	58.0/38.4	-/33.2	-	-	Residue-aware CF + GrabCut scale refinement
TCTrack [51]	2022	CNN	81.2/62.2	72.5/53.0	79.9/59.4	80.0/60.5	61.9/45.7	132.7/-	8.8	9.7	Temporal context modeling
DRCI [52]	2023	CNN	81.4/61.8	84.0/59.0	83.4/60.0	76.7/59.7	62.0/47.6	268.5/61.0	3.6	8.8	Contrastive instance representation learning

**Table 7:** The literature-reported benchmark overview of representative ViT-based trackers evaluated on UAV tracking benchmarks. “P/S” denotes Precision/Success, and “G/C” denotes GPU/CPU FPS; missing entries are marked by “-”. Values should be interpreted according to the rules in Section 5.

Method	Year	Arch	DTB70	UAVDT	VisDrone	UAV123	WebUAV-3M	FPS	FLOPs	Params	Efficiency Strategy
			(P/S)	(P/S)	(P/S)	(P/S)	(P/S)	(G/C)	(G)	(M)	
SGDViT [53]	2023	ViT	78.5/60.4	65.7/48.0	72.1/52.1	75.4/57.5	61.3/45.7	107.3/-	11.3	23.3	Saliency-guided dynamic tokens
Aba-ViTrack [15]	2023	ViT	85.9/66.4	82.9/59.1	86.1/65.3	86.4/66.4	67.4/53.7	172.1/46.3	2.4	8.0	Background-aware token pruning
AVTrack [16]	2024	ViT	84.3/65.0	82.1/58.7	86.0/65.3	84.8/66.8	69.0/54.0	252.8/53.8	0.97–1.9	3.5–7.9	Adaptive depth/dynamic computation
Aba-ViTrack-SA [15,43]	2025	ViT	86.1/65.2	83.1/59.4	87.7/65.7	86.0/67.9	70.7/55.5	188.4/47.9	2.4	8.0	Token pruning with self-adaptive update
AVTrack-SA [16,43]	2025	ViT	85.5/64.8	82.8/58.3	86.6/65.4	85.8/67.2	71.2/55.3	268.0/55.3	0.97–1.9	3.5–7.9	View-invariant adaptive computation

**Table 8:** The literature-reported benchmark overview of representative generic deep tracking baselines frequently used as reference methods in UAV tracking evaluation. “P/S” denotes Precision/Success. Missing entries are marked by “-”. The FPS column reports GPU FPS values reproduced from later UAV tracking comparative studies. Values should be interpreted according to the rules in Section 5.

Method	Venue	Main Benchmark	Metric Style	Prec.	Succ.	GPU FPS
DiMP18 [9]	CVPR 2019	DTB70	Precision + FPS	79.8	-	73.0
DiMP50 [9]	CVPR 2019	DTB70	Precision + FPS	79.2	-	52.4
PrDiMP18 [35]	CVPR 2020	DTB70	Precision + FPS	84.0	-	55.7
PrDiMP50 [35]	CVPR 2020	DTB70	Precision + FPS	76.4	-	42.1
SiamMask [54]	CVPR 2019	DTB70	Precision + FPS	76.9	-	109.6
SiamRPN++ [7]	CVPR 2019	DTB70	Precision + FPS	79.9	-	58.2
TransT [10]	CVPR 2021	DTB70	Precision + FPS	83.6	-	53.7
KeepTrack [55]	ICCV 2021	DTB70	Precision + FPS	83.6	-	19.5
SparseTT [56]	IJCAI 2022	DTB70	Precision + FPS	82.3	-	31.5
SiamGAT [57]	CVPR 2021	DTB70	Precision + FPS	75.1	-	92.3
OSTrack [12]	ECCV 2022	VisDrone2018	Prec/Succ/FPS	84.2	64.8	62.7
AQATrack [58]	CVPR 2024	VisDrone2018	Prec/Succ/FPS	87.2	66.9	53.4
HIPTrack [59]	CVPR 2024	VisDrone2018	Prec/Succ/FPS	86.7	67.1	31.3
ROMTrack [60]	ICCV 2023	VisDrone2018	Prec/Succ/FPS	86.4	66.7	51.1
EVPTrack [61]	AAAI 2024	VisDrone2018	Prec/Succ/FPS	84.5	65.8	22.1
SeqTrack [62]	CVPR 2023	VisDrone2018	Prec/Succ/FPS	85.3	65.8	15.3
MAT [63]	CVPR 2023	VisDrone2018	Prec/Succ/FPS	81.6	62.2	68.4
SAOT [64]	ICCV 2021	VisDrone2018	Prec/Succ/FPS	76.9	59.1	35.4
MixFormerV2 [65]	NeurIPS 2023	UAVDT	Prec/Succ/FPS	57.8	42.1	248.0
SimTrack [66]	ECCV 2022	UAVDT	Prec/Succ/FPS	76.5	57.2	76.0
ZoomTrack [67]	NeurIPS 2023	UAVDT	Prec/Succ/FPS	77.1	58.0	62.3

**Table 9:** Performance overview of Mamba-family and recent state-space-oriented UAV trackers. Benchmarks, metric order, and numerical results follow the corresponding source papers and should be interpreted according to Section 5.

Method	Year	Reported Benchmarks	Metric Order	Key Reported Results
TrackingMamba [23]	2024	OTMJ/UAV123/DTB70	AUC/Precision	OTMJ: 65.54/87.39; UAV123: 68.70/89.80; DTB70: 66.20/86.00
TrackingMiM [68]	2025	DTB70/UAVDT/VisDrone2018/UAV123/UAV123@10fps	Precision/Success	DTB70: 86.7/67.8; UAVDT: 85.0/62.4; VisDrone2018: 86.8/66.2; UAV123: 87.1/68.0; UAV123@10fps: 86.1/67.1
SAMViTrack [43]	2025	DTB70/UAVDT/VisDrone2018/UAV123/WebUAV-3M	Precision/Success	DTB70: 83.3/63.8; UAVDT: 82.1/58.1; VisDrone2018: 84.1/63.6; UAV123: 81.9/64.1; WebUAV-3M: 70.2/54.6
MambaNUT [69]	2025	NAT2024-1/NAT2021/UAVDark135	Precision/normalized precision/Success	NAT2024-1: 83.3/76.9/63.6; NAT2021: 70.1/64.6/52.4; UAVDark135: 70.0/69.3/57.1
MSTFT [27]	2026	UAV123/UAV123@10fps/UAV20L	AUC/Precision	UAV123: 79.4/84.5; UAV123@10fps: 76.5/84.1; UAV20L: 75.8/83.6
SPM-Track [70]	2026	DTB70/UAV123/UAV20L/UAVTrack112/LaSOT	Success/Precision	DTB70: 67.5/88.3; UAV123: 70.3/91.5; UAV20L: 70.2/92.2; UAVTrack112: 72.0/88.3; LaSOT: 69.8/75.1
LF-SSM [71]	2026	UAV123/VisDrone2018/ARDMAV/LaSOT	UAV123, VisDrone2018, ARDMAV: AUC/Precision; LaSOT: AUC/Normalized Precision/Precision	UAV123: 73.2/92.5; VisDrone2018: 69.2/88.5; ARDMAV: 67.8/86.2; LaSOT: 75.8/85.5/84.2

**Table 10:** Model efficiency statistics of recent Mamba-family UAV trackers with reported efficiency values. The table summarizes the literature-reported parameters, FLOPs, and speed values rather than providing a unified efficiency ranking. CPU-only and embedded edge-platform speeds are distinguished in parentheses.

Method	Params (M)	FLOPs (G)	GPU Speed (FPS)	CPU/Edge Speed (FPS)
TrackingMiM [68]	-	-	268.3	97.2 (CPU)
SAMViTrack [43]	4.0	1.1	314.1	61.6 (CPU)
MambaNUT [69]	4.1	1.1	72.0	-
MSTFT [27]	70.0	26.8	45.0	-
SPM-Track [70]	20.8	8.0	141.7	42.5 ± 1.9 (Jetson AGX Orin, edge platform)
LF-SSM-S [71]	18.5	12.8	320.0	69.0 (Jetson Orin Nano, TensorRT FP16, edge platform)

**Table 11:** Recommended minimum reporting checklist for deployment-oriented UAV tracking evaluation.

Reporting Item	Why It Matters for UAV Tracking
Hardware platform	FPS and latency depend strongly on whether evaluation uses a desktop GPU, CPU, Jetson-class device, or other embedded platform.
Input resolution and search-region size	These settings directly affect token count, memory usage, FLOPs, and small-target visibility.
Precision mode and inference stack	FP32, FP16, TensorRT, ONNX Runtime, custom CUDA kernels, and framework-level optimizations can lead to substantially different speeds.
Batch size and online setting	UAV tracking is normally an online batch-size-one task; throughput measured under batched inference may overstate deployment speed.
Warm-up, repeated runs, and latency statistics	Mean FPS alone can hide initialization overhead, tail latency, and unstable runtime behavior.
Memory footprint	Onboard deployment is constrained not only by computation but also by GPU/CPU memory and buffer usage during long sequences.
Power or thermal mode, when available	Sustained UAV operation can be affected by power limits, thermal throttling, and device frequency changes.
Accuracy–efficiency operating points	Matched curves or clearly defined operating points are more informative than a single best FPS or accuracy number.

## Data Availability

No new data were created or analyzed in this study. Data sharing is not applicable to this article.
